# Fabrication of Bifunctional Chitosan-Based Flocculants: Characterization, Assessment of Flocculation, and Sterilization Performance

**DOI:** 10.3390/ma11102009

**Published:** 2018-10-17

**Authors:** Moxi Wang, Li Feng, Xiaowei Fan, Dongmei Li, Wenqi Qu, Shuxian Jiang, Shaoxiu Li

**Affiliations:** 1 Key Laboratory of the Three Gorges Reservoir Region’s Eco-Environment, Ministry of Education, Chongqing University, Chongqing 400045, China; wmx0215_cqu@163.com (M.W.); fxw1014@126.com (X.F.); qwq2000@163.com (W.Q.); 2 School of Civil and Transportation Engineering, Guangdong University of Technology, No. 100, Waihuan Xi Road, Higher Education Mega Center, Panyu District, Guangzhou 510006, Guangdong, China; ldm108@163.com (D.L.); jiangshuxian_gdgy@163.com (S.J.); Water@gdut.edu.cn (S.L.)

**Keywords:** chitosan-based flocculant, bifunctional, turbidity removal, sterilization, plasma initiation

## Abstract

In this study, a series of chitosan-based quaternary ammonium graft flocculants, namely chitosan-*graft*-poly(acrylamide and methacryloyl ethyl trimethyl ammonium chloride) [CTS-*g*-P(AM-DMC)], was successfully synthesized by plasma initiation, and the as-prepared [CTS-*g*-P(AM-DMC)] had both flocculation and sterilization functions. Various characterization techniques were used to study the structure and physicochemical properties of the chitosan-based flocculants. ^1^H nuclear magnetic resonance spectroscopy (^1^H NMR), Fourier transform infrared spectroscopy (FTIR), X-ray diffraction spectroscopy (XRD), and thermogravimetric analysis/differential scanning calorimetry (TG/DSC) confirmed the successful synthesis of CTS-*g*-P(AM-DMC). Scanning electron microscopy (SEM) analysis exhibited that CTS-*g*-P(AM-DMC) contained a smooth convex and porous structure with an enormous surface area. CTS-*g*-P(AM-DMC) was then used to flocculate the simulated wastewater of the kaolin suspension and the *Salmonella* suspension. Besides external factors, such as the dosage of flocculant and pH, the effect of the internal factor graft ratio was also evaluated. The experimental results showed that CTS-*g*-P(AM-DMC) also revealed a strong sterilization effect, aside from the excellent flocculation effect. Moreover, the sterilization mechanism was investigated through a series of conductivity measurements and the analysis of fluorescence-based cell live/dead tests. The results indicated that CTS-*g*-P(AM-DMC) destroyed the cell membrane of *Salmonella* through its grafted quaternary ammonium salt, thereby exhibiting sterilization property.

## 1. Introduction

Water is an indispensable material resource for humans to survive, live, and produce. In the face of the current severe water pollution situation, it is imperative to develop efficient and environmentally-friendly water treatment technologies.

Flocculation is one of the most widely used economical and effective technologies for wastewater treatment, and the flocculant that is used has a great influence on the flocculation process [1,2]. There is no doubt that the development of flocculants with the characteristics of being economical, highly efficient, and environmentally-friendly is an irresistible trend, which has splendid application prospects [3]. Suspended particles and pathogenic microorganisms are usually the main pollutants in raw water, and are always treated by conventional treatment processes including coagulation/flocculation, precipitation, filtration, and sterilization in most water treatment plants in China and other countries [4,5,6]. In fact, the removal of turbidity and microorganisms are two separate processing units. The suspended particles can be effectively removed in the flocculation process by flocculants, and disinfectants are used to effectively kill the most pathogenic microorganisms [7]. However, wastewater discharge is increasing every year, and continues to expand with the population explosion as well as the rapid development of industry and the economy. The wastewater mainly contains suspended solid, organic substances and pathogenic bacteria such as *Salmonella* and *Escherichia coli*, which severely pollutes the water environment and deteriorates the water quality [8]. Therefore, in order to comply with the increasingly stringent national drinking water hygiene and quality standards, the wastewater must be disposed of with larger doses of flocculants and disinfectants in traditional ways. This not only means higher processing costs, but also increases the amount of secondary pollution and poses potential health risks to humans through elements such as the toxic disinfection by-products of halogen-containing disinfectants [9,10]. In view of the various deficiencies in the traditional water treatment process, associating coacervation/flocculation manipulation with disinfection features is an environmentally-friendly, economical, and effective way to fabricate flocculating agents with the dual functions of flocculation and sterilization. However, investigations into this issue are exiguous.

While inorganic and organic polymer flocculants have revealed universality and high efficiency in the flocculation course in the past few decades, some of these flocculants have often caused new environmental issues and secondary pollution to the environment, including for instance, the problem of the surplus metal ions of inorganic flocculants, toxic residual monomers of synthetic organic polymer flocculants, and so on [11,12,13]. In addition, it has also been reported that conventional inorganic flocculants and synthetic organic polymer flocculants have no significant disinfection activity [7,14]. Therefore, compared to traditional flocculants, natural organic polymers and their modified flocculants have become a hot spot of research due to their high efficiency, low cost, and biodegradability. Among them, chitosan is a deacetylated outcome of chitin that contains a large amount of free hydroxyl and amino groups in the molecular chain [15]. The chitosan molecular chains that are positively charged under acidic conditions are more favorable for flocculation, as most of the inorganic suspension particles and pathogenic microorganisms in water contain negative surface charges [16]. Obviously, this basic specialty of chitosan affords conditions for the removal of negatively charged suspension particles and bacteria. Moreover, it has been reported that chitosan exhibits antibacterial and sterilization capacity to a certain extent [17,18]. As a result of its firm intermolecular and intramolecular hydrogen bond, chitosan can be dissolved merely in acidic solutions. Namely, under such acidic conditions, chitosan is partially degraded, leading to a greatly reduced molecular weight and a feasible reduction of flocculation efficiency [19,20,21]. Furthermore, the cationic performance of the primary amino groups of chitosan is not adequately strong, and can be greatly reduced in the case of excessive nutrients and salts [16,22]. Therefore, it is necessary and imperative to overcome these disadvantages and enhance its flocculation performance via chemical modification. Fortunately, the introduction of quaternary ammonium groups with a strong hydration capacity and greater resistance into chitosan not only immensely weakens the intermolecular hydrogen bonds to increase the water solubility of the chitosan derivatives, it also improves its molecular weight and positive charge as well as its premier antibacterial capabilities [19,23]. Additionally, the hanging branches on the flocculant skeleton make the water contaminants more accessible and adsorbable [24,25]. The research on flocculants synthesized on the basis of chitosan has become more and more accessible, and these prepared flocculants based on chitosan have been used in heavy metal removal and sludge dewatering [26,27] However, few studies have reported on the synthesis of chitosan-based flocculants with a dual function, and their wide application needs to be verified in practice [7].

Based on the above considerations, a chitosan-based flocculant, chitosan-*graft*-poly(acrylamide-and methacryloyl ethyl trimethyl ammonium chloride) (CTS-*g*-P(AM-DMC)) with both bactericidal and flocculation functions was synthesized. CTS-*g*-P(AM-DMC) was prepared by plasma-initiated grafting copolymerization using a plasma simplex energy source to irradiate monomers for a short period, before the reactants were placed at an appropriate temperature for polymerization [28]. Plasma-initiated polymerization is environmentally-friendly and sustainable; thus, it eliminates secondary pollutants from external initiators, avoids reagent pollution, prepares linear products, and operates simply [29,30]. As a result, plasma-initiated polymerization has been proposed for the graft copolymerization of chitosan. Fourier transform infrared spectroscopy (FTIR), ^1^H nuclear magnetic resonance spectroscopy (^1^H NMR), and X-ray diffraction spectroscopy (XRD) were used to characterize the structure of CTS-*g*-P(AM-DMC) and verify the successful synthesis of the polymer. In addition, the morphology and thermal decomposition capabilities of the polymer were observed by SEM and thermogravimetric analysis/differential scanning calorimetry (TG/DSC). Next, the flocculation and bactericidal properties of CTS-*g*-P(AM-DMC) were systematically studied. Kaolin and *Salmonella*, which are ubiquitous in actual wastewater, were used as pollutants in the synthetic effluents. It is worth emphasizing that *Salmonella* was first used for the sterilization of chitosan-based flocculants. Moreover, the flocculation and sterilization mechanisms were investigated and discussed in detail.

## 2. Materials and Methods

### 2.1. Materials

Acrylamide (AM) was of industrial grade and purchased from the Chongqing Lanjie Tap Water Company (Chongqing, China); methacryloyl ethyl trimethyl ammonium chloride (DMC) (50 wt % in water, industrial grade), chitosan (CTS) (≥95% degree of deacetylation, 100–200 mPa·s viscosity), and fluorescein isothiocyanate (FITC) were obtained from Shanghai Aladdin Biochemical Science Stock Company Ltd. (Shanghai, China), which were of analytical grade; *Salmonella typhimurium* (AS1.1174) was provided by the Institute of Microbiology, Chinese Academy of Sciences; propidium (PI), Mueller–Hinton agar (MHA), and Mueller–Hinton broth medium (MHB) were purchased from Shanghai Yeasen Biotech Co., Ltd. (Shanghai, China), solvent and analytical reagents were all of analytical grade; kaolin was sourced from Chongqing Chuandong Chemical Industry Co., Ltd. (Chongqing, China). For the control test, polyaluminum chloride (PAC) and disinfectant 1231 were purchased from Kelong Chemical Reagent Co., Ltd. (Chengdu, China). The water that was used in all of the experiments was deionized water. All of the analytical grade reagents were used without further purification.

### 2.2. Synthesis of Copolymers

**Scheme 1:**
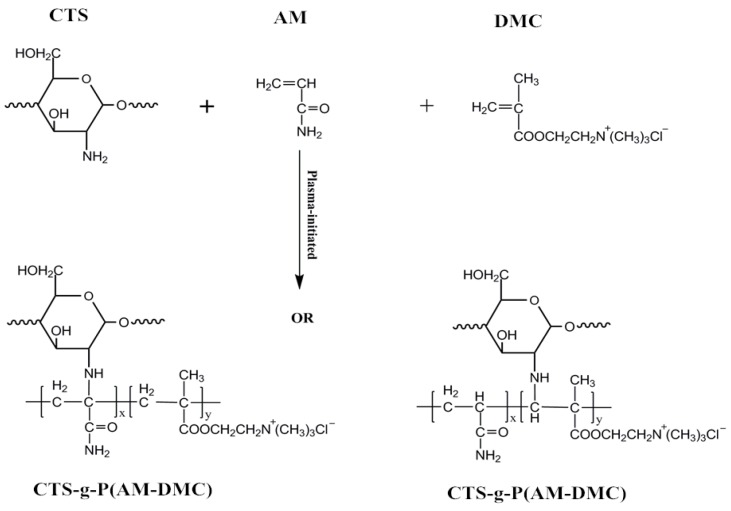
The synthesis of chitosan-*graft*-poly(acrylamide-and methacryloyl ethyl trimethyl ammonium chloride) (CTS-*g*-P(AM-DMC)).

In order to have a clear understanding of the reaction process, the plasma-initiated graft copolymerization reaction is shown in Scheme 1 [28]. 

The vinyl chemical bond is split up under plasma radiation to generate the original monomer AM or DMC free radicals, and the AM or DMC free radicals combine and attack the adjacent DMC and AM monomers to form a relatively large polymer; this process is called chain propagation. When the DMC and AM monomers were consumed completely, the chain termination reaction took place, and the copolymer of P(AM-DMC) was obtained. Meanwhile, the deprotonated amino group in chitosan is prone to react with the electrophile of the newly formed P(AM-DMC) to facilitate successful grafting and the formation of the final product, CTS-*g*-P(AM-DMC) [31]. A given mass of CTS was dissolved in 10 mL of 1.0% dilute acetic acid with slow stirring. A preordained amount of AM, DMC, and deionized water were then added into the chitosan aqueous solution with steady stirring until entirely dissolved, and pure N_2_ was filled for 15 min to completely drive off the oxygen. Next, the homogeneous reaction solution was energized and initiated in the plasma device (13.56 MHz, SY300W, Institute of Microelectronics, Chinese Academy of Sciences, Beijing, China) with a certain discharge time and power. Then, the mixture was shaken in a water bath shaker at a suitable post polymerization temperature for a certain period of time. The polymerizates were purified in acetone and ethanol. Finally, the obtained solid polymer was dried to a constant weight in a vacuum oven at 60 °C. 

### 2.3. Characteristics of Copolymers

The CTS-*g*-P(AM-DMC) flocculants were characterized by ^1^H NMR, FTIR, XRD, SEM, and TG/DSC, respectively. The ^1^H NMR and FTIR spectra of the polymers were conducted with an Avance-500 NMR spectrometer (Bruker Company, Karlsruhe, Germany) with standard pulse procedures in deuterium oxide (D_2_O) and a 550-Series II infrared spectrometer (Burker Company, Zurich, Switzerland) using KBr (Potassium bromide) as pellets, respectively. XRD patterns of the polymers were obtained by an X-ray diffractometer (SmartLabTM 3 KW, Tokyo, Japan) equipped with graphite monochromatized Cu Kα radiation (λ = 1.54056 Å). The morphological features of CTS-*g*-P(AM-DMC) were inspected by a MIRA 3 LMU SEM (TES-CAN, Brno, Czech Republic). TG/DSC was performed on a synchronal thermal analyzer (DTG-60H, Shimadzu, Japan) to inspect the thermal stability of copolymers under a nitrogen atmosphere from a temperature of 20 °C to 600 °C. In this section, chitosan (CTS), a copolymer of AM and DMC (P(AM-DMC)), and CTS-*g*-P(AM-DMC) were selected for the comparative analysis.

### 2.4. Bacterial Culture

All of the experiments were performed under gnotobasis. The media solution and glassware were autoclaved at 121 °C for 30 min prior to the microbiological experiments. A small amount of *Salmonella* was scraped from the strains of inclined plane, and then the cells were prepared by culturing in MHB at 37 °C for 16 h.

### 2.5. Preparation of Simulated Wastewater

A total of 1.0 g of kaolin powder was added to 1.0 L of water to obtain a 0.1 wt % kaolin suspension under ultrasonic dispersion. The cells from the *Salmonella* culture were assembled by being centrifuged at 3000 *g* for 5 min, and were then diluted with PBS (0.01 M, pH 7.3) to achieve approximately 1 × 10^7^ CFU/mL for preparation of the bacterial suspension. Finally, the prepared bacterial suspension was stored in a refrigerator at 4 °C for later use.

### 2.6. Flocculation Experiment

The flocculation tests were implemented on a program-controlled jar test apparatus (ZR4-6, Zhongrun, China) at room temperature. Furthermore, the details of the flocculants that were used for the flocculation experiment are listed in Table 1, where the intrinsic viscosities of the polymers were measured by one-point methods [32]. The flocculant storing solution was freshly prepared by dissolving 0.1 g of flocculant in 100 mL of water before each experiment. The pH of the synthetic effluent was adjusted by 0.1 mol/L HCl and 0.1 mol/L NaOH; then, a certain amount of CTS-*g*-P(AM-DMC) stock solution was added to the synthetic effluent with a stirring at 200 rpm for 5 min until completely mixed and followed by slow stirring at 50 rpm for 15 min to strengthen the flocculant growth. Then, it was settled without stirring for 30 min to keep the flocculation equilibrium. Supernatants at a depth of 2 cm below the water surface of these sample solutions were collected for further analysis. Each survey was implemented in triplicate, and the end results were the average of three runs. In addition, the relative error was controlled to less than 5.0%. The analytical methods for turbidity and *Salmonella* removal rates are illustrated in Supporting Text S1. In addition, the bactericidal mechanism of CTS-*g*-P(AM-DMC) was also investigated by measuring the conductivity of the solution by using a conductivity meter and recording the fluorescence-based cell live/dead tests using a fluorescence microscope (Olympus IX71, Tokyo, Japan). The measurement method for conductivity is shown in Supporting Text S2.

## 3. Result and Discussion

### 3.1. ^1^H NMR Spectrum

**Figure 1:**
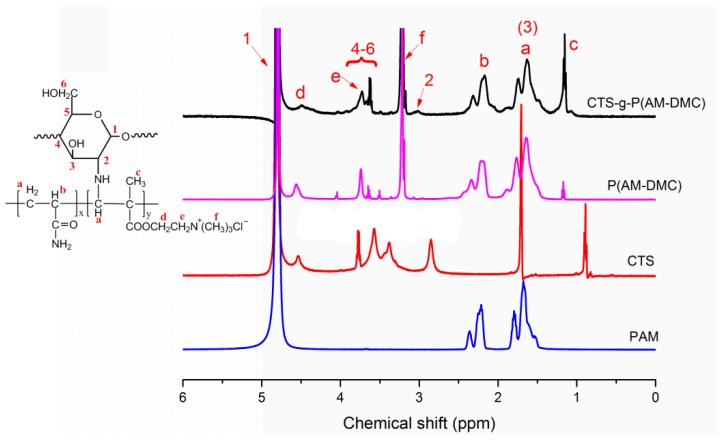
^1^H nuclear magnetic resonance spectroscopy (^1^H NMR) spectra of PAM, chitosan (CTS), P(AM-DMC), and CTS-*g*-P(AM-DMC).

The ^1^H NMR spectrum of CTS-*g*-P(AM-DMC) and the corresponding distribution of the signals are shown in Figure 1. Compared with the spectra of PAM (Polyacylamide), P(AM-DMC), and CTS, each corresponding peak in the CTS-*g*-P(AM-DMC) spectrum showed a certain shift on account of the changes in the chemical microenvironment. The proton signals of the chitosan skeleton were revealed at 4.79 ppm of the H_(1)_ proton, 3.02 ppm of the H_(2)_ proton, 1.63 ppm of the H_(3)_ proton, and 3.42–3.73 ppm of the H_(4–6)_ proton in the CTS-*g*-P(AM-DMC) spectrum when compared with those displayed in CTS [33]. Corresponding to the AM monomer, the asymmetric peaks at 1.63 ppm for H_(a)_ and 2.17 ppm for the H_(b)_ proton were dated from the resonances of –CH_2_– and methine –CH–, respectively [34]. The peaks of the H_(c)_, H_(d)_, H_(e)_, and H_(f)_ protons at 1.17 ppm, 4.49 ppm, 3.73 ppm, and 3.21 ppm originating from –CH_3_, –O–CH_2_–, –CH_2_–N^+^, and –(CH_3_)_3_ in the DMC monomer were observed in the CTS-*g*-P(AM-DMC) spectrum, respectively. Therefore, the ^1^H NMR spectroscopy analytical results verified the successful graft copolymerization of AM, DMC, and CTS.

### 3.2. FTIR Spectral Analysis

**Figure 2:**
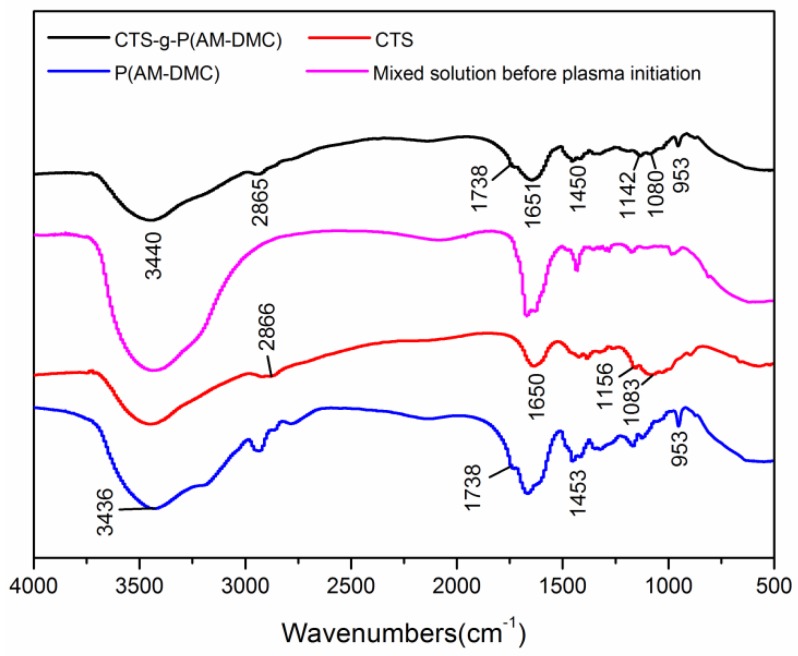
Fourier transform infrared spectroscopy (FTIR) spectra of CTS-*g*-P(AM-DMC), CTS, P(AM-DMC), and the mixture solution.

To affirm the molecular structure of the synthetic flocculants, the FTIR spectrum of CTS-*g*-P(AM-DMC), CTS, P(AM-DMC), and their mixture were obtained and analyzed in Figure 2.

As shown in the spectrum of CTS, the absorption peaks at 2866 cm^−1^, 1650 cm^−1^, 1156 cm^−1^, and 1083 cm^−1^ were attributed to the stretching vibrations of the carbon–hydrogen bond (C–H) that arose from the methyl group (–CH_3_), the bending vibration of the primary amine (N–H), the asymmetric deformation vibration of C–O–C, and the stretching vibration of the primary alcohol (C–OH), respectively [28,35]. At the same time, the peaks around 1738 cm^−1^, 1453 cm^−1^, and 953 cm^−1^ were shown in the FTIR spectrum of P(AM-DMC). These three peaks were the characteristic peaks of the DMC unit, and attributed to the ester group, ethylene group, and quaternary ammonium methyl, respectively [36]. Furthermore, the wide characteristic absorption peak around 3436 cm^−1^ was attributed to the stretching vibration of the –NH_2_ groups in the AM unit [34]. Most of the characteristic absorption peaks of P(AM-DMC) and CTS occurred in the FTIR spectra of CTS-*g*-P(AM-DMC), and the FTIR spectra for the mixture of AM, DMC, and CTS differed from those in the CTS-*g*-P(AM-DMC) spectrum. Based on a comparative analysis, these spectra further verified the successful synthesis of CTS-*g*-P(AM-DMC). Furthermore, the absorption peaks for the skeleton structure of CTS remained invariant after grafting on P(AM-DMC), demonstrating that grafting copolymerization would not influence the internal skeleton structure of CTS molecules.

### 3.3. XRD Patterns

**Figure 3:**
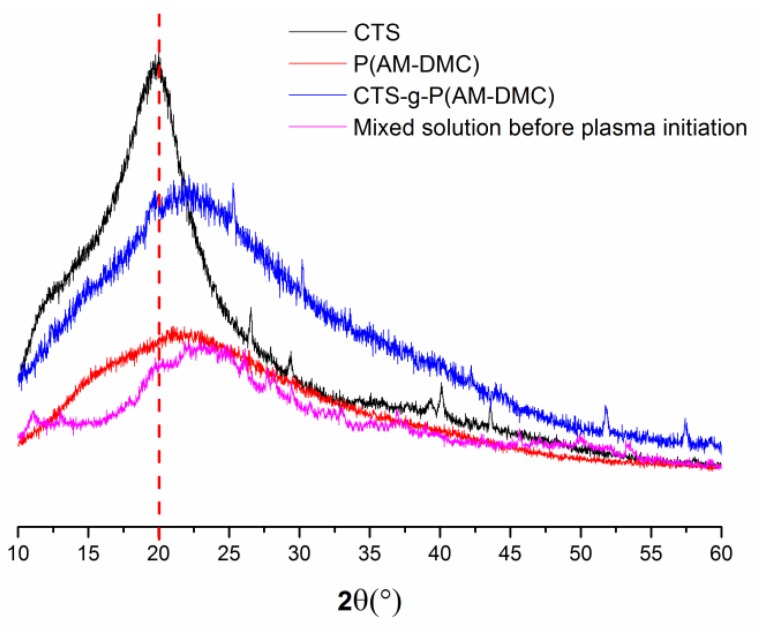
X-ray diffraction spectroscopy (XRD) patterns of CTS, P(AM-DMC), CTS-*g*-P(AM-DMC), and the mixture solution.

The XRD spectra of CTS, P(AM-DMC), CTS-*g*-P(AM-DMC), and their mixture are shown in Figure 3. A strong characteristic diffraction peak displayed at 2θ = 20° in the spectrum of CTS was assigned to the crystalline form II of chitosan. Given the obviously higher crystallinity of CTS than that of P(AM-DMC), the characteristic diffraction peak of P(AM-DMC) was wider and weaker than CTS [37]. Compared with the XRD spectrum of CTS, the characteristic diffraction peak of CTS-*g*-P(AM-DMC) after grafting copolymerization became weaker and wider, and a right shift appeared. Due to the introduction of the branched chain, resulting in the reduction of the overall structural order in the polymer, the crystallinity of CTS-*g*-P(AM-DMC) was lowered [38]. This indicated that the hydrogen bond capacity of chitosan was decreased after the grafting of AM and DMC onto the chitosan backbone. Nevertheless, compared with P(AM-DMC), the containment of CTS with high crystallinity in CTS-*g*-P(AM-DMC) was enhanced, and narrowed its characteristic peak. Hence, the results of XRD spectroscopy offered further evidence for the successful synthesis of CTS-*g*-P(AM-DMC). The XRD patterns of the mixture of AM, DMC, and CTS differed from those in the CTS-*g*-P(AM-DMC) and CTS, and indicated the formation of CTS-*g*-P(AM-DMC) from another perspective. Moreover, CTS-*g*-P(AM-DMC) had an amorphous structure rather than a crystalline structure as CTS; therefore, the solubility of CTS-*g*-P(AM-DMC) became higher than that of CTS.

### 3.4. TG/DSC Analysis

**Figure 4:**
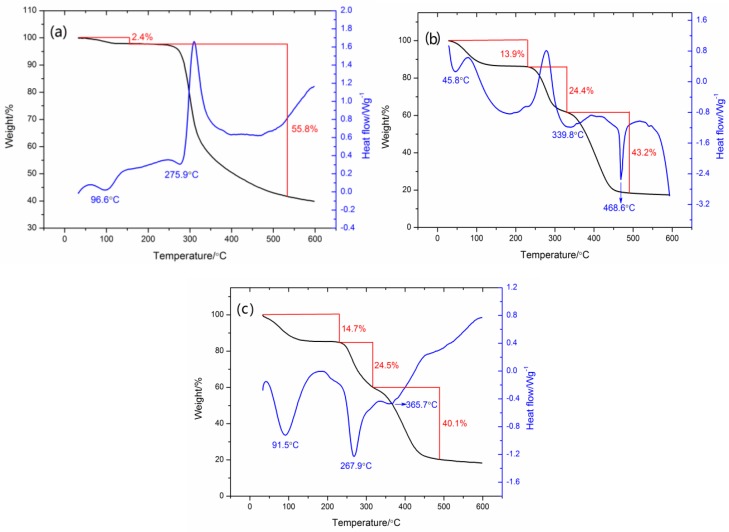
Thermogravimetric curves of (**a**) CTS, (**b**) P(AM-DMC), and (**c**) CTS-*g*-P(AM-DMC).

Figure 4 displays the thermal gravimetric curves of (a) CTS, (b) P(AM-DMC), and (c) CTS-*g*-P(AM-DMC). The TG and DSC represent the thermogravimetry and differential scanning calorimetry, respectively.

As shown in Figure 4c, the TG curve of CTS-*g*-P(AM-DMC) displayed three stages of weight loss. The first stage of weight loss (14.7%) existed between 33 °C and 230 °C, which could be attributed to the loss of adsorption and the evaporation of combined water. The second weight loss occurred in the range of 230–316 °C with a weight loss of 24.5%, corresponding to the imine reaction of the amide group and the deprivation of methyl and dehydrochlorination from the quaternary ammonium group (–C(CH_3_)_3_N^+^Cl–) [27]. The weight loss in the third stage occurred between 316–490 °C, with a weight loss of 40.1%, which was due to the thermal decomposition of the chitosan skeleton [39]. The copolymer decomposed entirely at about 490 °C. Beyond 490 °C, the TG curve flattened without any weight loss. The ultimate weight of the residue was 20.7% of the original weight of the flocculant sample. The reason for the weight of P(AM-DMC) in Figure 4b was similar to that of CTS-*g*-P(AM-DMC) in Figure 4c. Accordingly, the three endothermic peaks were shown at 91.5 °C, 267.9 °C, and 365.7 °C in the CTS-*g*-P(AM-DMC) DSC curves, respectively. The TG curve of CTS only had two stages of weight loss in Figure 4a when compared with CTS-*g*-P(AM-DMC). Additionally, the endothermic peak in the DSC curve of CTS at 275.9 °C could be discovered in the DSC curve of CTS-*g*-P(AM-DMC) at 267.9 °C. Furthermore, in the DSC curve of P(AM-DMC), the endothermic peak of the second stage weight loss was observed at 365.7 °C in the DSC curve of CTS-*g*-P(AM-DMC). Therefore, the features of the TG curves for CTS and P(AM-DMC) were found in the TG curve of CTS-*g*-P(AM-DMC). All proof and discussions of thermal gravimetric characterization proved the successful synthesis of CTS, AM, and DMC. The TG/DSC results also indicated that CTS-*g*-P(AM-DMC), after the copolymerization of CTS, AM, and DMC, had good thermal stabilities, which are more favorable for its storage and application at room temperature without any extra freezing measures.

### 3.5. SEM of Polymers

**Figure 5:**
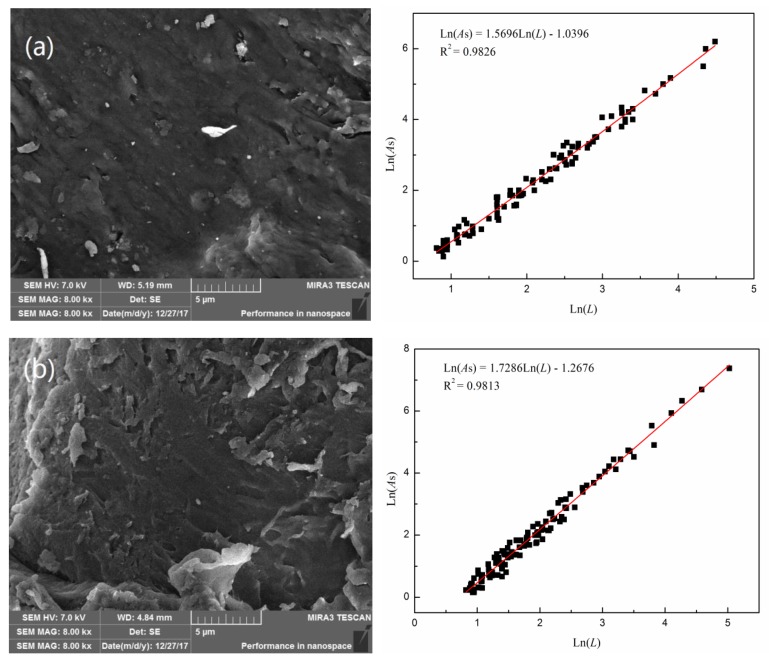

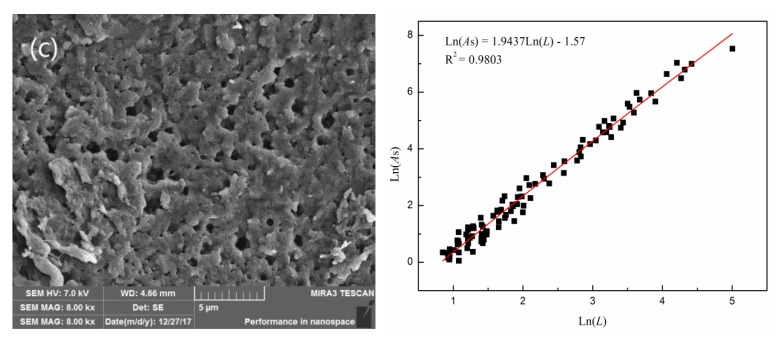
Morphology analysis of (**a**) CTS, (**b**) P(AM-DMC), and (**c**) CTS-*g*-P(AM-DMC).

The SEM images of CTS, P(AM-DMC), and CTS-*g*-P(AM-DMC) are displayed in Figure 5. Obviously, the surface morphologies of these three flocculants varied differently. According to Figure 5a,b, the surface of CTS was relatively smooth without convex and pore-like structures [19]. P(AM-DMC) was composed of a layer structure with a relatively flat and bumpy surface morphology. Apparently, after P(AM-DMC) was grafted onto the CTS backbone, the surface morphology of CTS-*g*-P(AM-DMC) was significantly different from that of P(AM-DMC) and CTS. As shown in Figure 5c, CTS-*g*-P(AM-DMC) contained a smooth convex and porous structure with an enormous surface area [33]. Under the effect of the flocculant surface activation and the generation of free radicals caused by plasma action, the introduction of the cationic groups into the CTS backbone weakened and destroyed the primitive ordered crystal structure of CTS. The chemical bond formed by the reaction of free radicals and monomer radicals increased the flocculant surface roughness; thereby, an increase in the surface porosity was observed. The changes in the surface morphology also indicated that AM and DMC were successfully grafted onto the CTS backbone by a plasma-initiated method. Apart from observing the SEM images directly, the two-dimensional fractal dimension (*D*_2_) of the flocculants were calculated from the SEM images by Image-Pro Plus 6.0 software illustrated in Supporting Text S3, and the results are shown in Figure 5. The average *D*_2_ of CTS and P(AM-DMC) was 1.5696 and 1.7286, respectively. However, the average *D*_2_ of CTS-*g*-P(AM-DMC) was 1.9437, which was much higher than the other two flocculants, indicating that it had a more chaotic surface morphology. The porous and chaotic structure of CTS-*g*-P(AM-DMC) observed by SEM is more useful and facile to contact with water to increase its water solubility in a short time. More and more soluble polymer chains will extend and stretch in water to catch and trap the colloidal particles; therefore, the flocculation performance is greatly enhanced. The flocculation test results of CTS-*g*-P(AM-DMC) in Section 3.6.1 and Table 2 were in keeping with the surface morphology analyses in this section.

### 3.6. Evaluation of Dual Function Performance: Flocculation and Sterilization

**Figure 6:**
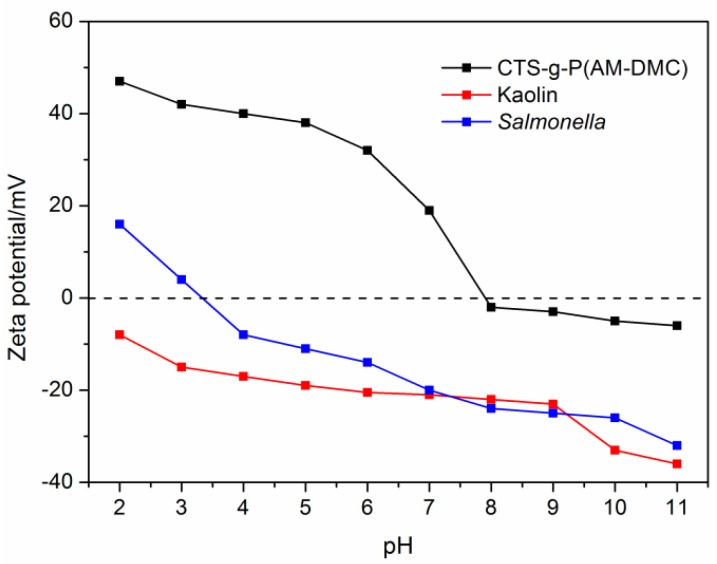
Zeta potential (ZP) vs. pH profiles of CTS-*g*-P(AM-DMC), kaolin suspension, and *Salmonella* suspension.

The dual function of CTS-*g*-P(AM-DMC) was systematically evaluated using a kaolin suspension and *Salmonella* suspension as synthetic wastewater. Due to the important effect of the charge neutralization of the flocculant on the flocculation performance and mechanism, zeta potential (ZP) values of the flocculant, the synthetic wastewater, and their pH dependence were measured in Figure 6.

The measurement method for the zeta potential is shown in Supporting Text S4. The positive electrical property of CTS-*g*-P(AM-DMC) was significantly enhanced due to the introduction of strong cationic quaternary ammonium salt groups onto the CTS skeleton. Additionally, CTS-*g*-P(AM-DMC) revealed the typical charge properties of amphoteric polyelectrolytes [7]. A positive charge was seen at pH ≤ 7.9, while the ZP became negative at pH > 7.9. In addition, the kaolin suspension contained a negative surface charge over the measured pH range, while the *Salmonella* suspension contained a negative surface charge when the pH > 3.4.

#### 3.6.1. Flocculation Performance of Kaolin Suspension

**Figure 7:**
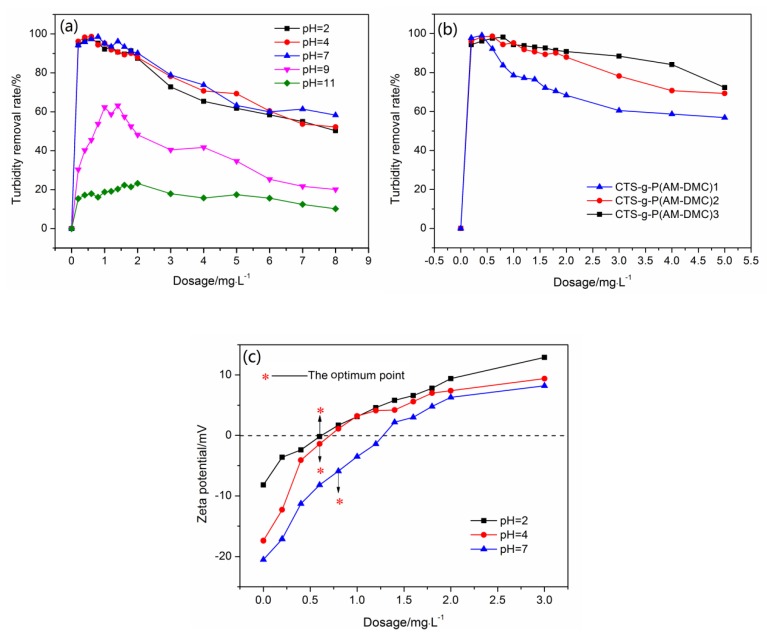
Flocculation performance of CTS-*g*-P(AM-DMC) in the kaolin suspension: (**a**) Effect of dosage and pH; (**b**) Effect of graft ratio; (**c**) Zeta potential of the supernatant as a function of flocculants dosages.

The flocculation performance of flocculants is influenced by some external factors. Among them, the dose of the flocculant and the pH of the wastewater are two vital factors [40]. Hence, the influence of CTS-*g*-P(AM-DMC) dosage on the flocculation of the kaolin suspension at several tested pH values was studied in Figure 7a. Figure 7a shows that the removal rate of turbidity of CTS-*g*-P(AM-DMC) was high, and the corresponding optimal dosage of the flocculant was relatively low under acidic and neutral conditions. However, the contaminant removal rate was much lower under alkaline conditions. This diversity could be explained by the synergistic effects of charge and conformation: under the acidic or neutral condition, CTS-*g*-P(AM-DMC) was rich in positive charges on account of the grafted quaternary ammonium salt group; thereby, this chitosan-based flocculant had a stronger charge neutralization ability and exhibited good charge attraction for the negatively charged kaolin particles. On the other hand, due to the electrostatic repulsion of the flocculant and the extended macromolecular conformation on the acidic condition, the flexible grafted P(AM-DMC) branch on the CTS-*g*-P(AM-DMC) comb copolymer greatly enhanced the flocculant chain expression, which was favorable for the bridging as well as the flocculation process [7,9]. The extended macromolecular conformation attributed to the intramolecular electrostatic repulsion of the flocculant greatly enhanced the accessibility between the flocculant and the suspended colloid particles [3]. Therefore, more and more kaolin particles were intercepted and anchored on the polymer chain and its branch to form large and compact flocculants. Nevertheless, these effects were reversed under alkaline conditions, so the acidic condition was more rewarding toward the flocculation process.

Meanwhile, the graft ratio as a structural factor is one of the most important factors for graft copolymers; thus, the effect of the grafting rate of CTS-*g*-P(AM-DMC) on the flocculation performance was also systematically evaluated in Figure 7b. According to Figure 7b, it was observed that the flocculation performance for the flocculants showed a similar trend of increasing first to a maximum value, and then decreased slowly at all of the flocculant dosages. This trend was in accordance with the typical behavior of the polyelectrolytes in the flocculation process that was reported in previous studies [19,36]. At the outset, the negatively charge kaolin particles destabilized and were collected due to the charge neutralization and bridging, and the turbidity removal rate of the supernatant increased accordingly. Whereas, when the flocculant was applied excessively, the excess flocculant was adsorbed on the flocculant surface to result in the repulsion of positive charge agglomerates of the flocculants and caused their restabilization [41].

As shown in Figure 7c, the ZPs displayed a growing trend with the increased flocculant dose at different pH values. Thus, simple charge neutralization and patching were the primary flocculation mechanisms except for the bridging effect [25]. At pH 2.0 and pH 4.0, the zeta potential for CTS-*g*-P(AM-DMC) were close to zero at their optimum doses, and was less than zero at pH 7, demonstrating that simple charge neutralization was predominant at pH 2.0 and 4.0, but patching contributed more when the pH was further increased. Under acidic conditions, CTS-*g*-P(AM-DMC) with a high positive charge density greatly attracted the kaolin particles so that the surfaces of the kaolin particles were completely and rapidly coated by the flocculant, thereby completing the charge neutralization [7,42]. While it was in the neutral condition, the conformation of CTS-*g*-P(AM-DMC) began to disintegrate and the surface charge decreased, leading to the reduction of adsorption ability and the uneven surface coating of kaolin particles. At a higher pH, the exposed surface portion of a negatively charged particle might adhere to another particle with a positively charged flocculant before complete coverage, resulting in the patching and bridging flocculation mechanism.

The optimal doses and homologous turbidity removal rates at several tested pH conditions are summarized in Table 2. The flocculation performance of the widely used inorganic flocculant PAC for the flocculation of kaolin suspensions was also carried out at different pH values for further contrast. It was observed from Table 2 that at high pH conditions, the corresponding optimal doses of CTS-*g*-P(AM-DMC) increased slightly with increasing pH, which indicated that an excellent flocculation performance would be obtained at a lower dosage. Moreover, as the concentration of the anti-ionic (OH^−^) around the polymer chains increased, the positive charge on the surface decreased, and the repulsive force of the molecular chains weakened, causing the polymer chains to shrink and the adsorption ability for the kaolin particles to be decreased [19]. In contrast, the PAC mainly worked through the effects of charge neutralization, sweeping, and netting, and more flocculants were required to obtain a desired purification effect at higher pH conditions. It was shown in Table 2 that CTS-*g*-P(AM-DMC) had a higher flocculation efficiency and lower optimal dose than PAC. This phenomenon could be attributed to CTS-*g*-P(AM-DMC) having a macropore and porosity structure, and therefore more effective flocculation sites on its surface as well as an increased bridging effect [16].

#### 3.6.2. Flocculation and Sterilization Performance of *Salmonella* Suspension

After a long period of study, it was discovered that there were many resemblances between flocculants and disinfectants, especially in functional groups. Both quaternary ammonium salt disinfectants and the tested flocculants contain a mass of quaternary ammonium groups. Quaternary ammonium compounds can be utilized as bactericides by charge attraction, osmosis, and diffusion [19,43]. In wastewater, most of the suspended colloidal particles and bacteria are negatively charged, hence chitosan-based flocculants with quaternary ammonium groups should likewise contain a certain bactericidal effect. Therefore, it has been predicted that the synthesized CTS-*g*-P(AM-DMC) possessed dual functions of flocculation and sterilization. Thus, the flocculation and sterilization properties of CTS-*g*-P(AM-DMC) in the *Salmonella* suspension were systematically evaluated to verify this conjecture.

**Figure 8:**
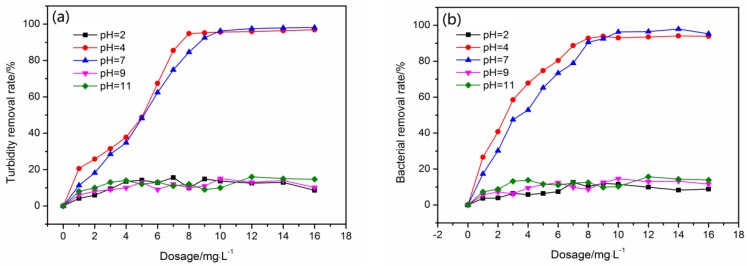

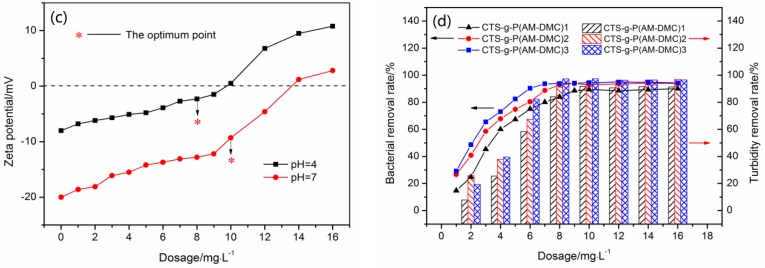
Flocculation and sterilization performance of CTS-*g*-P(AM-DMC) in *Salmonella* suspension: (**a**,**b**) Effect of dosage and pH; (**c**) Zeta potential of the supernatant as a function of flocculants dosages; (**d**) Effect of graft ratio.

Equally, the dose of the flocculant, the pH of the *Salmonella* suspension, and the graft ratio were the vital factors in the evaluation tests. Figure 8a,b show the changes in the turbidity removal rate and bacterial removal rate of CTS-*g*-P(AM-DMC) with the flocculant dosage at different pH conditions, which revealed a very different tendency from the restabilization of the flocculant during the flocculation of the kaolin suspension. When the flocculant was overdosed, the turbidity removal rate–dose curves reached a plateau without a restabilization process, which was in contrast to the traditional restabilization. Hence, apart from the single effects of charge neutralization, bridging, and netting, it could be speculated that there was a synergistic effect during the process. In addition, according to Figure 8a,b, the flocculant showed significant high turbidity removal efficiency and bacterial removal efficiency at pH 4 and pH 7, but was much lower at pH 2, pH 9, and pH 11 due to electrostatic repulsion. Based on the ZP measurement shown in Figure 6, CTS-*g*-P(AM-DMC) exhibited opposite electrical properties with the *Salmonella* suspension in the pH range of 3.3–7.9, so CTS-*g*-P(AM-DMC) could successfully exert the charge neutralization effect. However, the electrostatic repulsion was dominant when the electrical property of the CTS-*g*-P(AM-DMC) and *Salmonella* suspension were the same charge beyond the pH range of 3.3–7.9. Therefore, charge attraction might be at least one of the major flocculation mechanisms.

Furthermore, according to Table 2, the optimum dose of CTS-*g*-P(AM-DMC) for the flocculation of the *Salmonella* suspension increased as the pH increased, which was similar to that of the kaolin suspension. As the pH increased, the ZP of *Salmonella* was more negative, while that of CTS-*g*-P(AM-DMC) was less positive, thus weakening the charge attraction and increasing the flocculant dosage. It was further compared with the *Salmonella* flocculation performance conditioned by CTS-*g*-P (AM-DMC), PAC, and disinfectant 1231 in Table 2. In the tested *Salmonella* suspension, CTS-*g*-P(AM-DMC) showed better flocculation behavior than that of PAC. This might be due to the porous structure with a large specific surface area of the CTS-*g*-P(AM-DMC) microstructure and its longer polymer chain for improving the bridging effect. As a disinfectant, 1231 exhibited the best bactericidal properties as a professional bactericidal disinfectant, but showed the worst flocculation performance because of its inferior bridging and netting effect, while CTS-*g*-P(AM-DMC) displayed a relatively significant sterilization property. The above analysis confirmed that the synthesized CTS-*g*-P(AM-DMC) possessed excellent flocculation and sterilization properties.

Moreover, according to the change in the final supernatant ZP flocculated with CTS-*g*-P(AM-DMC) at pH = 4 and pH = 7 in Figure 8c, the ZP at the optimum dose was negative. Therefore, the patching effect made a contribution to the flocculation of the *Salmonella* suspension. Figure 8d showed the dose dependence of the turbidity removal rate and the bacterial removal rate of CTS-*g*-P(AM-DMC) with different graft ratios on the *Salmonella* suspension. The graft ratio of P(AM-DMC) in CTS-*g*-P(AM-DMC) was studied. Among the three tested chitosan-based flocculants, as the graft ratio of P(AM-DMC) increased, the corresponding turbidity removal rate gradually increased. The CTS-*g*-P(AM-DMC)3 with the highest graft ratio and the largest surface positive charge exhibited the highest flocculation efficiency due to its enhanced electrical neutralization and bridging. As the introduction of quaternary ammonium ions strengthened the charge neutralization and sterilization property of CTS-*g*-P(AM-DMC)3, the turbidity removal rate and the bacterial removal rate reached their plateau quickly, which further provided a basis for the existence of another mechanism.

## 4. Sterilization Mechanism in *Salmonella* Suspension

**Figure 9:**
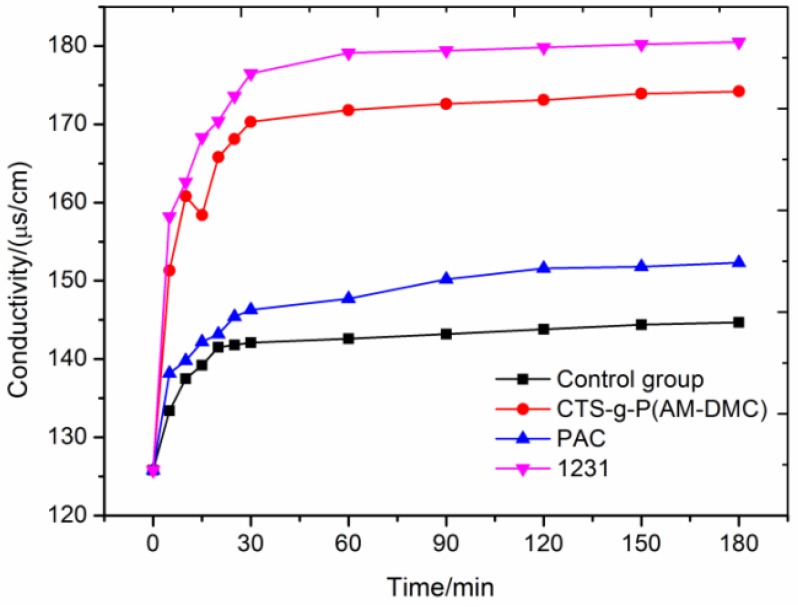
Conductivity of the *Salmonella* suspension as a function of time.

In order to provide an in depth study of the flocculation mechanism, conductivity measurements and the fluorescence-based cell live/dead tests of bacterial cells were performed. The variation of conductivity is an important indicator of the bacterial cell structure change. The *Salmonella* cell membrane rupture can be determined based on the changes in conductivity, and the sterilization mechanism of CTS-*g*-P(AM-DMC) in the *Salmonella* suspension could be further explored. Figure 9 shows the change in conductivity of the *Salmonella* suspension under the action of CTS-*g*-P(AM-DMC). It can be concluded that the conductivity of the *Salmonella* suspension without flocculants showed a slight increase, whereas the conductivity change trends of the *Salmonella* suspension flocculated with PAC and without a flocculant were almost the same, indicating that the *Salmonella* cell membranes were not damaged. As a result, PAC possessed a weak sterilization effect. The minimal bacterial removal rate of PAC in the *Salmonella* suspension could be ascribed to its flocculation effect. However, after adding 1231 and CTS-*g*-P(AM-DMC) as disinfectants, the conductivity of the *Salmonella* suspension was significantly increased. The curve trend of the CTS-*g*-P(AM-DMC) sample was similar to that of the 1231 sample. The conductivity after treatment with CTS-*g*-P(AM-DMC) rapidly reached 151 μS/cm in 5 min, indicating that CTS-*g*-P(AM-DMC) caused a certain degree of damage to the *Salmonella* cell membrane. When the membrane permeability changed, intracellular ions such as charged intracellular polymeric substances, K^+^, Ca^2+^, and Mg^2+^ were excreted, causing an increase in the conductivity in the *Salmonella* suspension [44]. This affected the normal growth and metabolism of the cells, and ultimately led to the death of the *Salmonella*. Consequently, the sterilization effect of CTS-*g*-P(AM-DMC) was not just antibacterial, but in fact bactericidal.

**Figure 10:**
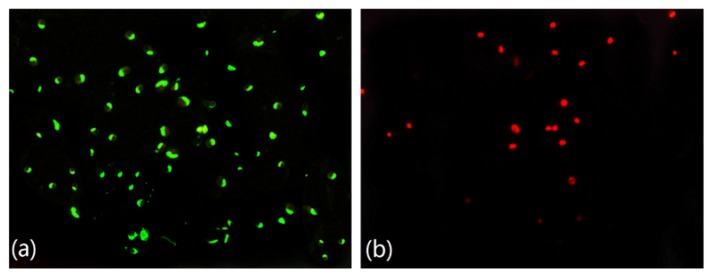

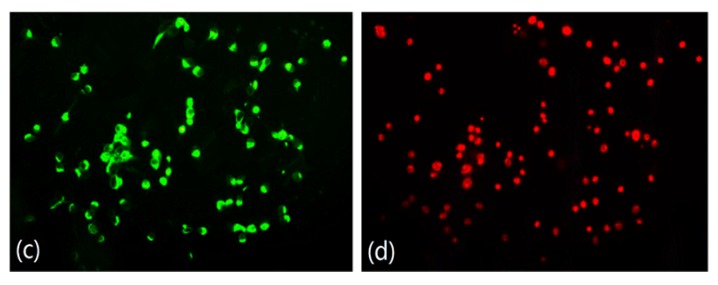
Fluorescent images of live/dead *Salmonella*: (**a**,**b**) Blank control group; (**c**,**d**) Treatment with CTS-*g*-P(AM-DMC).

To confirm the sterilization effect of CTS-*g*-P(AM-DMC), the fluorescent-based cell live/dead tests of bacterial cells were subsequently measured in Figure 10.

The *Salmonella* cells were stained by FITC and PI, which are fluorescent nucleic acid dyes. Among them, FITC is a cell-penetrating dye with a green fluorescence that can mark both live and dead cells, while PI is a cell-impervious dye with a red fluorescence that can only mark dead cells [45]. Figure 10a,b illustrate that there were only a few dead cells in the blank control group. In contrast, after flocculation with CTS-*g*-P(AM-DMC) (displayed in Figure 10c,d), the *Salmonella* cells showed an observably stronger red fluorescence, indicating that the cytomembranes of *Salmonella* were impaired and marked by PI. This significant difference indicated that CTS-*g*-P(AM-DMC) had a remarkable sterilization performance through the attack and destruction of the *Salmonella* cell membrane. This result was concordant with the changes in the conductivity of the *Salmonella* suspension.

**Figure 11:**
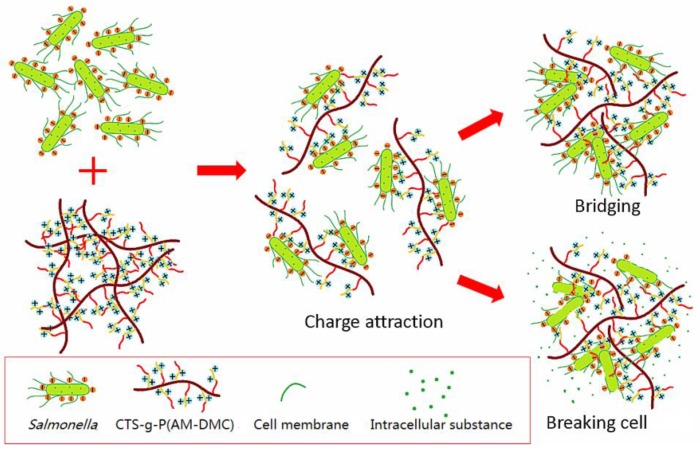
Flocculation and sterilization mechanism of CTS-*g*-P(AM-DMC).

Combining all of the above flocculation studies, including the conductivity measurements and the fluorescence-based cell live/dead tests of *Salmonella* together, it could be qualitatively speculated that the *Salmonella* cell membrane was impaired after flocculation with high efficiency; hence, its normal metabolism and mass transfer were undermined, and the ultimate death of the bacteria took place. The flocculation of *Salmonella* by CTS-*g*-P(AM-DMC) was different to the flocculation of inorganic suspended colloid particles due to the sterilization performance of the quaternary ammonium groups, as well as the enhanced charge attraction and bridging effects resulting from the P(AM-DMC) grafting of CTS. The proposed flocculation mechanism in *Salmonella* suspension is depicted in Figure 11.

First, the negatively charged *Salmonella* was adsorbed on CTS-*g*-P(AM-DMC) due to the intense charge adsorption; then, the repulsion between the cells decreased and the cells destabilized. Next, due to the bridging action of the excess flexible P(AM-DMC) branches in CTS-*g*-P(AM-DMC), the flocculants aggregated together to form larger and denser flocculants. Finally, CTS-*g*-P(AM-DMC) destroyed the cell membrane by the quaternary ammonium groups on the P(AM-DMC) branch; hence, intracellular substances were released. In summary, the synthesized CTS-*g*-P(AM-DMC) revealed both effective flocculation and sterilization effects. The dual function can be attributed to the ability as follows. The CTS-*g*-P(AM-DMC) attracted and aggregated *Salmonella* through strong electrostatic interactions, and then destroyed its cell membrane effectively through the quaternary ammonium group, where it also intercepted *Salmonella* through bridging and netting effects using its flexible P(AM-DMC) branch.

## 5. Conclusions

In this study, a chitosan-based bifunctional flocculant CTS-*g*-P(AM-DMC) was synthesized by plasma-initiated graft copolymerization, and the success of its graft copolymerization was proven by the characterization analytical results of ^1^H NMR, FTIR, XRD, SEM, and TG/DSC. Furthermore, CTS-*g*-P(AM-DMC) was used to flocculate kaolin and *Salmonella* suspensions. All of the results of the flocculation tests revealed that CTS-*g*-P(AM-DMC) possessed excellent flocculation effects and an effective sterilization effect under mildly acidic and neutral conditions. Based on the measurements of the zeta potentials, for the flocculation of kaolin suspensions, charge neutralization was dominated under acidic conditions. However, patching made a greater contribution under neutral conditions. Nonetheless, the patching effect of *Salmonella* was more pronounced under all of the effective pH conditions. The flocculation properties of CTS-*g*-P(AM-DMC) were strengthened with the increase of the graft ratio, which encouraged the significance of the charge attraction. According to the results of the conductivity measurements and fluorescence-based cell live/dead tests, it was qualitatively inferred that the cell membrane of *Salmonella* was destroyed by the CTS-*g*-P(AM-DMC) with grafted quaternary ammonium branches through its enhanced electrical neutralization and bridging effect. Since CTS-*g*-P(AM-DMC) contained dual functions of flocculation and sterilization, it is expected that the dosage of the disinfectant that is required in water treatment plants could be significantly reduced, thus resulting in a conspicuous cost reduction and avoiding the risk of secondary contamination by the disinfection by-products. It has important significance for the actual water treatment project. Although the current preparation cost of chitosan-based flocculants is relatively high due to the high price of chitosan and the energy consumption during preparation, its long-term and large-scale applications will show significant advantages once its low-cost preparation technology is roundly developed.

## Figures and Tables

**Table 1:** The details of the flocculants used for the flocculation experiment.

Flocculants ^a^	Grafting Ratio (%)	Intrinsic Viscosity (dL·g^−1^)
P(AM-DMC)	/	6.27
CTS-*g*-P(AM-DMC) 1	92	6.56
CTS-*g*-P(AM-DMC) 2	173	6.13
CTS-*g*-P(AM-DMC) 3	235	6.50
PAC	/	/
1231	/	/

^a^ CTS-*g*-P(AM-DMC): the three kinds of CTS-*g*-P(AM-DMC) with different grafting ratios were named as CTS-*g*-P(AM-DMC) 1, CTS-*g*-P(AM-DMC) 2 and CTS-*g*-P(AM-DMC) 3, respectively; P(AM-DMC): copolymer of acrylamide (AM) and methacryloyl ethyl trimethyl ammonium chloride (DMC); PAC: polyaluminum chloride (content >30%); 1231: lauryl trimethyl ammonium chloride. The calculation of grafting ratio was illustrated as that in the previous study [27].

**Table 2:** Flocculation performance of CTS-*g*-P(AM-DMC), PAC, and 1231 at different pH.

	pH	Kaolin		*Salmonella*		
		Optimal Dose (mg/L)	Turbidity Removal Rate (%)	Optimal Dose (mg/L)	Turbidity Removal Rate (%)	Bacterial Removal Rate (%)
CTS-*g*-P(AM-DMC)	2	0.6	97.8	—	—	—
	4	0.6	98.6	8	94.8	92.8
	7	0.8	98.5	10	96.3	96.3
PAC	2	2	91.6	—	—	—
	4	4	97.2	20	58.1	39.6
	7	5	97.9	23	59.8	39.4
1231	2	—	—	—	—	—
	4	—	—	8	7.4	92.1
	7	—	—	10	8.5	97.8

## Supplementary Materials

The following Supporting text are available online at http://www.mdpi.com/1996-1944/11/10/2009/s1. Text S1. Analytical methods for turbidity removal rate (%) and bacterial removal rate (%). Text S2. Measurement methods for conductivity. Text S3. Fractal dimensions of CTS-g-P(AM-DMC), P(AM-DMC) and CTS. Text S4. Measurement methods for Zeta potential.
