# Supplementary material for: Fabrication of Bifunctional Chitosan-Based Flocculants: Characterization, Assessment of Flocculation, and Sterilization Performance
Source: Materials (Basel). 2018 Oct 17;11(10):2009. doi: 10.3390/ma11102009 (PMC6213324; doi:10.3390/ma11102009)
Supplement: Supplementary file 1 [file materials-11-02009-s001.pdf]

*Supplementary Materials:*

# Fabrication of Bifunctional Chitosan-Based Flocculants: Characterization, Assessment of Flocculation, and Sterilization Performance

**Moxi Wang [1,2], Li Feng [1,2,*], Xiaowei Fan [1], Dongmei Li [2], Wenqi Qu [1], Shuxian Jiang [2] and Shaoxiu Li [2]**

[1] Key Laboratory of the Three Gorges Reservoir Region's Eco-Environment, Ministry of Education, Chongqing University, Chongqing 400045,China; wmx0215_cqu@163.com (M.W.); fxw1014@126.com (X.F.); qwq2000@163.com (W.Q.)

[2] School of Civil and Transportation Engineering, Guangdong University of Technology, No. 100, Waihuan Xi Road, Guangzhou, Higher Education Mega Center, Panyu District, Guangzhou 510006, Guangdong, China; ldm108@163.com (D.L.);jiangshuxian_gdgy@163.com (S.J.); Water@gdut.edu.cn (S.L.)

* Correspondence: fl19860314@126.com; Tel./Fax: +86-135-9461-7802

**Text S1.** Analytical methods for turbidity removal rate (%) and bacterial removal rate (%)

(1) Measurement of turbidity removal rate (%)

The supernatant at 1 cm below the surface after flocculation was collected with a syringe, and its turbidity was measured by using a turbidimeter (2100Q, HACH) to observe the flocculation performance of CTS-g-P(AM-DMC) in the purified synthetic test suspension. The turbidity removal rate is defined in Equation (1):

$$\text{Turbidity removal rate (\%)} = \frac{T_1 - T_0}{T_1} \quad (1)$$

where $T_1$ and $T_0$ are the turbidity of the supernatants before and after flocculation, respectively.

(2) Measurement of bacterial removal rate (%)

The flocculation property of CTS-g-P(AM-DMC) for the purification of *Salmonella* suspension depended on the transmittance of the synthetic test suspension before and after the flocculation experiment using an ultraviolet spectrophotometer (TU-1901, Beijing Purkinje General Instrument Co., Ltd., Beijing, China) with a wavelength of 600 nm. The removal rate can be calculated by Equations (2) and (3):

$$OD_{600} = \lg \frac{1}{T} \quad (2)$$

$$\text{Bacterial removal rate(\%)} = \frac{OD_{1(600)} - OD_{0(600)}}{OD_{1(600)}} \quad (3)$$

where $T$ is the transmittance of the tested supernatant, and $OD_{1(600)}$ and $OD_{0(600)}$ are the optical densities of the tested supernatant before and after flocculation, respectively.

**Text S2.** Measurement methods for conductivity

Set a blank sample, PAC sample and bactericide 1231 sample as the control group. In the jar test flocculation process, 5 mL samples were taken from each jar at 0, 5, 10, 15, 20, 25, 30, 60, 90, 120, 150 and 180 minutes respectively. Then the samples were centrifuge at 4000 g for 15

min, and their conductivity was measured by using a conductivity meter (DDS-307, Shanghai, China).The measurements were performed in parallel three times and the average was calculated.

**Text S3.** Fractal dimensions of CTS-g-P(AM-DMC), P(AM-DMC) and CTS

The fractal dimension (*D*) of polymers calculated by the SEM-based image analysis method can be used as supporting information for the SEM images to assess the surface morphology of polymers.

The two-dimensional fractal dimension ($D_2$) of CTS-g-P(AM-DMC), P(AM-DMC) and CTS was measured by SEM images analysis and calculated by Equation (4), in which $L$ and $A_s$ are the perimeter and projected area of selected region, respectively.

$$A_s = \mathrm{k} \times L^{D2} \tag{4}$$

$$\mathrm{Ln}(A_s) = D_2 \times \mathrm{Ln}(L) + \mathrm{Ln}(\mathrm{k}) \tag{5}$$

$A_s$ and $L$ were calculated from the SEM images of the flocculants using Image-Pro Plus 6.0 Software, and linear regression analysis of the graph was performed in order to obtain the line slope ($D_2$) which is the sample two-dimensional fractal dimension.

**Text S4.** Measurement methods for Zeta potential

The supernatant at 1 cm below the surface after flocculation was collected with a syringe, and its Zeta potential was measured by using a Zeta potential analyzer (Zetasizer Nano3000, U.K.). Each survey was implemented in triplicate and the end results were the average of three runs.
